# Baseline-referenced plasma metabolomic trajectories during early post-exercise recovery: A secondary analysis of public human exercise metabolomics data

**DOI:** 10.1371/journal.pone.0361757

**Published:** 2026-10-30

**Authors:** Yuanzhang Feng, Zhenwei Wu

**Affiliations:** School of Physical Education, Shanghai Normal University, Shanghai, China; University of California Riverside, UNITED STATES OF AMERICA

## Abstract

**Background:**

Post-exercise metabolomic changes are difficult to interpret without reference to the pre-exercise state. A baseline-referenced approach may help distinguish transient exercise responses from residual metabolic deviation during early recovery.

**Methods:**

We performed a secondary analysis of two public human plasma metabolomics datasets. ST001789 was used as the primary discovery dataset and included 57 paired plasma samples from 19 participants collected before exercise, immediately after a 90-min endurance run, and after 60 min of recovery. ST004303 was used as an external support dataset and included moderate-intensity and sprint-interval exercise conditions. We assessed paired baseline-referenced contrasts, recovery-pattern classes, residual baseline deviation, cross-dataset support, intensity-related behavior, and threshold sensitivity to prioritize candidate metabolites for future validation.

**Results:**

In ST001789, 198 metabolites differed from baseline immediately after exercise, 140 differed after 60 min of recovery, and 116 changed between the immediate post-exercise and recovery samples. Recovery-pattern classification showed heterogeneous early responses. Of 77 discovery features matched in ST004303, 74 had at least one direction-consistent external comparison and 66 had at least one FDR-supported external change. Staged prioritization identified 54 primary candidates. Corrected sensitivity schemes retained 136, 25, and 11 features, with 11 retained across all three schemes. These candidates spanned fatty-acid transport and oxidation, amino acid metabolism, lipid remodeling or signaling, and purine/nucleoside turnover.

**Conclusions:**

Baseline return, residual deviation, external support, and intensity-related behavior help organize early post-exercise metabolomic responses. The identified metabolites should be interpreted as hypothesis-generating candidates for future validation, not as validated recovery biomarkers.

## Introduction

Post-exercise recovery involves the gradual resolution of exercise-induced metabolic and physiological perturbations and supports subsequent adaptation [1,2]. In exercise science and sports practice, recovery monitoring is relevant to competitive sport, training prescription, rehabilitation-oriented exercise, and repeated-session planning, because practitioners need interpretable signals before a subsequent load is applied [3,4]. Current approaches commonly combine external workload, perceived exertion, wellness measures, heart-rate-derived variables, neuromuscular performance tests, and selected blood biomarkers [5–7]. However, these measures reflect different physiological dimensions and can be affected by testing context, sampling time, pre-analytical conditions, and individual baseline differences [6,7]. Plasma metabolomics may add a complementary biochemical perspective because circulating metabolites reflect substrate mobilization, mitochondrial energy turnover, amino acid and lipid remodeling, and systemic physiological stress [8–11]. Although exercise-metabolomics studies have described post-exercise changes in lipids, acylcarnitines, amino acids, tricarboxylic acid cycle intermediates, and purine-related metabolites [8–11], relatively few have organized these responses within a baseline-referenced framework that distinguishes immediate perturbation, residual deviation from baseline, and intensity-related behavior [11,12].

Acute exercise does not change circulating metabolites in a single pattern. Response magnitude and recovery kinetics vary across metabolites, reflecting energy demand, substrate mobilization, and post-exercise restoration. Early plasma metabolomics studies reported rapid changes in glycolytic intermediates, tricarboxylic acid cycle-related metabolites, nucleotide turnover products, lipolysis-related metabolites, and amino acid pathways, supporting blood metabolites as systemic indicators of the physiological response to exercise [13]. Time-resolved metabolomics and multi-omics studies have further shown that some metabolites return rapidly toward baseline, whereas others remain altered during recovery [14,15]. This heterogeneity is biologically plausible: acylcarnitines are linked to fatty-acid transport and mitochondrial fuel partitioning, purine-related metabolites to ATP turnover, and amino acid and lipid metabolites to substrate redistribution and stress-related remodeling [16–18]. Because these responses also vary with exercise mode, intensity, duration, participant characteristics, and environmental context, findings for individual metabolites may differ across studies [15,18–20]. This variability supports the need to evaluate post-exercise metabolite changes according to their reproducibility, interpretability, and behavior within a defined recovery window.

For interpreting post-exercise metabolomic responses, a metabolite is more informative when its change can be evaluated relative to the pre-exercise baseline. Such baseline-referenced profiles can show whether a metabolite returns toward baseline, remains altered during recovery, changes later in recovery, or responds more strongly to higher-intensity exercise. This distinction is relevant because the next exercise session is often planned before recovery is complete, when residual physiological stress may still affect neuromuscular function, perceived fatigue, and tolerance to repeated high-intensity work [21–23]. A baseline-referenced strategy can separate transient exercise responses from metabolites that show persistent deviation within a defined recovery window. It also allows recovery-related profiles to be compared across moderate-intensity and sprint-interval exercise, which may impose different recovery demands despite both being used to stimulate adaptation [22,24,25]. Candidate metabolites can therefore be prioritized not by statistical significance alone, but by recovery-pattern relevance, residual baseline deviation, cross-dataset consistency, and interpretable intensity-related behavior. These features should still be considered hypothesis-generating candidates, and their practical value must be tested against functional recovery, perceived exertion, and repeated-bout performance [26–28].

In the present study, we performed a secondary analysis of public human plasma metabolomics data to prioritize baseline-referenced metabolite candidates during early post-exercise recovery. Two publicly available human plasma metabolomics datasets from Metabolomics Workbench were analyzed [29]. ST001789 was used as the primary discovery dataset because it included paired samples collected before exercise, immediately after a 90-min endurance run, and 60 min into recovery [30,31]. ST004303 was used as an external intensity-support dataset because it included moderate-intensity and sprint-interval exercise conditions, allowing matched metabolites to be examined across different exercise loads [32]. We characterized immediate post-exercise responses, residual baseline deviation, recovery-pattern classes, cross-dataset support, intensity-related behavior, and threshold stability. We then combined these outputs to prioritize candidate metabolites for future recovery-focused validation. We expected that some circulating metabolites would show reproducible recovery-related behavior and interpretable intensity sensitivity, supporting their interpretation as hypothesis-generating candidates for prospective validation rather than as validated biomarkers.

## Materials and methods

### Study design and data sources

This study was a secondary analysis of two public human plasma metabolomics datasets from Metabolomics Workbench [29]. ST001789 was used to define baseline-referenced recovery profiles and included paired samples collected before exercise, immediately after a 90-min endurance run, and after 60 min of recovery [30,31]. ST004303 included moderate-intensity and sprint-interval exercise conditions [32] and was used to assess external exercise-related support for matched metabolites. Both datasets used plasma. The datasets were accessed on 26 May 2026 and 30 May 2026, respectively. The analysis aimed to prioritize candidate metabolites for future recovery-focused validation. Fig 1 summarizes the design, and Table 1 lists the analytical role of each dataset.

**Table 1:** Public metabolomics datasets and their analytical roles in the present study.

Dataset	Role	Exercise context	Sampling structure	Retained samples	Feature set	Main manuscript outputs
ST001789	Primary discovery dataset	Human plasma metabolomics after a 90-min endurance run	Pre, Time0 and Time60; paired baseline-referenced contrasts	19 participants; 57 samples (19 Pre, 19 Time0, 19 Time60)	689 raw features; 455 retained	Paired response analysis (198 Time0 vs Pre, 140 Time60 vs Pre, 116 Time60 vs Time0 FDR-supported metabolites); recovery-pattern classification; RBD; CERS 2.0 discovery evidence
ST004303	External intensity-support dataset	Human plasma metabolomics under MIE and SIE conditions	1P, before exercise; 2P, immediately after exercise completion; 4P, 3 h after exercise completion; same codes for MIE and SIE.	19 men; 57 samples (9 MIE participants / 27 samples; 10 SIE participants / 30 samples)	266 raw biological features; 266 retained; 77 ST001789 discovery features matched to ST004303 annotations	External support for ST001789-derived metabolites (74 direction-supported; 66 FDR-supported); EISI and IRCM candidate-prioritization outputs

Note: Values were extracted from Metabolomics Workbench metadata and corresponding analysis output files. Both datasets used plasma. FDR-supported indicates adjusted p < 0.05. All 57 ST004303 samples were collected during acute exercise testing before the eight-week training program. Sampling-code definitions and training status were confirmed by the corresponding author of the source study. Pre, pre-exercise baseline; Time0, immediately after exercise; Time60, 60 min into recovery; RBD, residual baseline deviation; CERS 2.0, study-specific staged consensus exercise-recovery score; EISI, exercise-intensity sensitivity index; IRCM, intensity–recovery coupling map; MIE, moderate-intensity exercise; SIE, sprint-interval exercise; FDR, false discovery rate.

**Fig 1:**
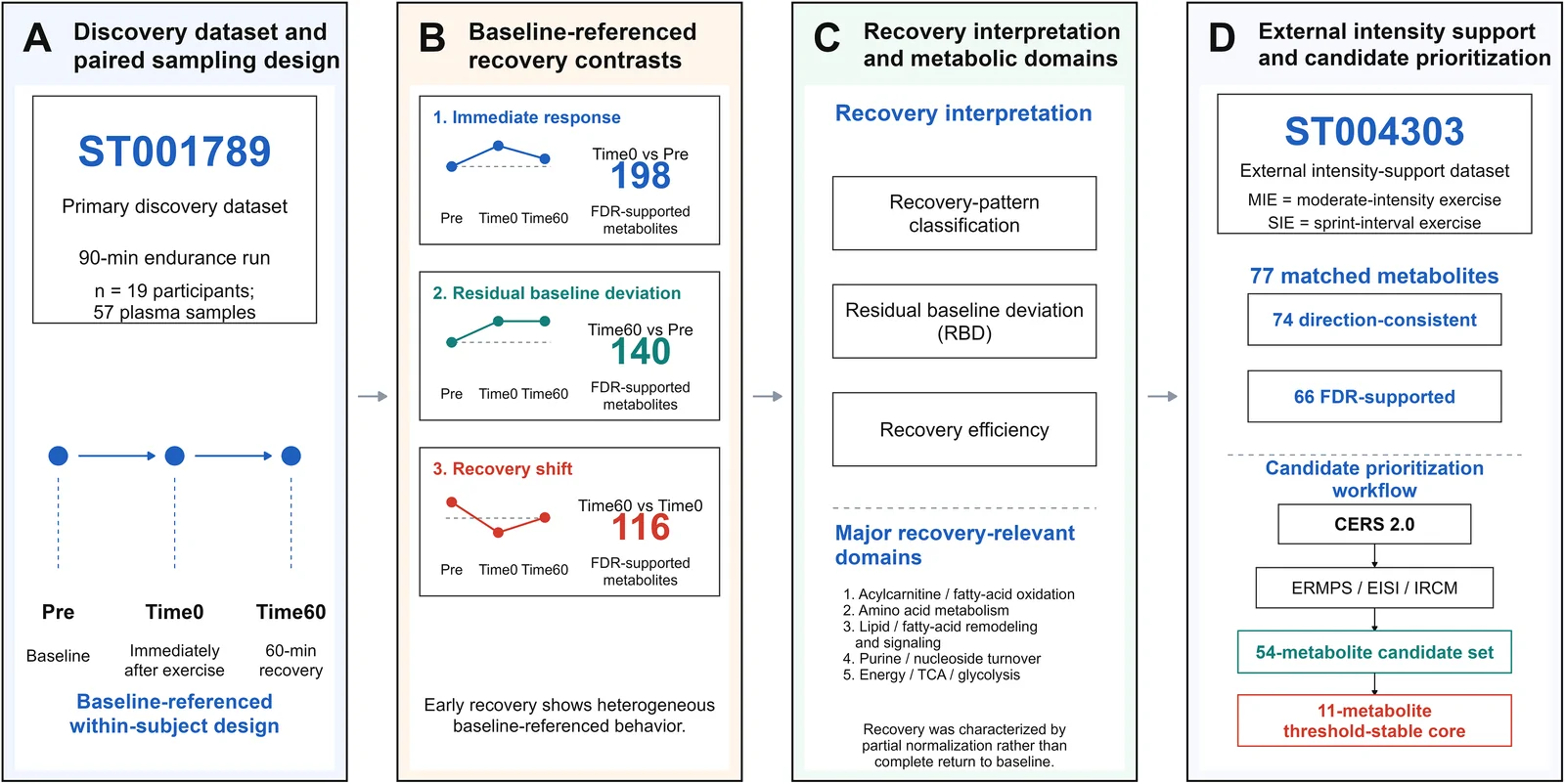
Study design and baseline-referenced exercise-recovery analysis. (A) ST001789 included paired plasma samples collected at Pre, Time0, and Time60 after a 90-min endurance run. (B) Baseline-referenced contrasts defined the immediate response, residual baseline deviation, and recovery shift, identifying 198, 140, and 116 FDR-supported metabolites, respectively. (C) Recovery interpretation combined pattern classification, residual baseline deviation, recovery efficiency, and recovery-relevant metabolic domains. (D) ST004303 provided external intensity support under MIE and SIE conditions; 77 matched metabolites yielded 74 direction-consistent and 66 FDR-supported signals. A staged prioritization procedure then identified a 54-metabolite candidate set and an 11-metabolite threshold-stable core for future validation. Abbreviations: CERS 2.0, study-specific staged consensus exercise-recovery score; EISI, exercise-intensity sensitivity index; ERMPS, exercise-recovery metabolite prioritization score; FDR, false discovery rate; IRCM, intensity–recovery coupling map; MIE, moderate-intensity exercise; SIE, sprint-interval exercise.

### Participant characteristics and sampling conditions

ST001789 included 19 young, physically active adults (13 men and 6 women; median age, 25 years). The original study reported fasting baseline blood collection and a morning run from 09:00–10:30, with recovery sampling at 11:30 [30]. Repository metadata additionally records a banana of approximately 100 g given after baseline sampling and before the run [31]. ST004303 included 19 men, with 9 participants in MIE and 10 in SIE. All 57 plasma samples were collected during acute exercise testing in the untrained state, before the eight-week training program. Sampling took place before exercise, immediately after exercise completion, and 3 h after exercise completion. The source protocol also describes provision of balanced meals during the preceding 24 h [32]. The different recovery windows and protocol conditions were considered when interpreting external support. The paired models included participant fixed effects; age, sex, fitness, nutritional intake, hydration, and clock time were not entered as additional covariates. Stable participant effects do not test sex-specific responses or remove time-varying confounding.

### Data preprocessing and metabolite annotation

ST001789 contained 689 named assay features across the HILIC and reversed-phase exports. The earlier count of 691 included one empty trailing row in each export. After excluding empty rows and features with more than 20% missing values, 455 features across 57 plasma samples remained. ST004303 contained 266 named features; 57 biological samples were retained after excluding quality-control and blank samples. Its feature-missingness threshold was 30%, followed by exclusion of zero-variance features; all 266 features remained. In each dataset, intensities were log2-transformed after adding half the minimum positive value in the retained matrix, then standardized within each feature across the retained samples. The code included feature-median imputation after transformation, but no values in either retained biological matrix required imputation. Participant identifiers and sampling times preserved the repeated-measures structure. Differential models used the standardized matrices; their coefficients therefore represent mean differences in feature-specific z-score units, not log2 fold changes.

### Metabolite identification and cross-dataset matching

Metabolite identities were taken from the deposited annotations; no new spectral identification was performed. For ST001789, the original investigators used broad-spectrum targeted LC-MS/MS with scheduled multiple reaction monitoring. Compound-specific transitions, fragmentation parameters, spectra, and retention times were optimized using purified standards [30]. ST004303 used HILIC-Orbitrap MS and data-dependent MS/MS of pooled quality-control material. The repository describes Compound Discoverer processing and retention of MS/MS library matches scoring at least 70 against mzCloud or MassBank of North America through mzVault [32]. These records support a distinction between a standard-informed targeted assay and library-supported annotations. They do not establish authentic-standard confirmation for every ST004303 feature. Structural or isomeric uncertainty in the source annotations therefore carries into the secondary analysis.

Matching used exact agreement between normalized feature-name or RefMet-name keys. The implementation converted names to lower case, removed “(low)” and “(high)”, replaced underscores with spaces, collapsed whitespace, and removed characters outside its specified alphanumeric and punctuation set. It also removed leading L-, D-, DL-, and optical-rotation prefixes and applied an explicit synonym dictionary covering selected organic acids and carnitines. Duplicate feature–key pairs were removed before joining. Each discovery-feature/external-feature/treatment/contrast combination was counted once, but one-to-many feature mappings were retained and aggregated. The complete normalization rules and synonym groups are documented in S1 Text and S1 File; S4 Table lists the reported matches and annotation flags. This permissive procedure matches reported annotations and does not confirm equivalent stereochemistry or resolve lipid isomers. For example, L-Carnitine matched both D-Carnitine and L-Carnitine, and L-Tryptophan matched D-(+)-Tryptophan. Broad biochemical modules did not determine differential-test significance, but did contribute one biological-interpretability point to ERMPS and were an eligibility condition in the sensitivity schemes.

### Baseline-referenced paired response analysis in ST001789

Within-participant responses in ST001789 were estimated using limma linear models with time and participant fixed effects (~0 + time + subject), followed by empirical Bayes variance moderation [33]. The model used feature-wise standardized log2 intensities. Three contrasts evaluated the immediate response (Time0–Pre), residual baseline deviation (Time60–Pre), and recovery-phase shift (Time60–Time0). Coefficients are standardized mean differences in z-score units. Benjamini–Hochberg adjustment was applied separately within each contrast across all 455 features; adjusted p < 0.05 defined statistical support. No additional covariates were included. Classification combined these tests with effect directions and relative baseline deviation; failure to detect a difference was not interpreted as an equivalence test.

### Recovery-pattern classification and residual baseline deviation

Recovery-pattern classes were assigned in the following order: neither baseline contrast significant, non-responsive; acute but not residual contrast significant, fast-recovering; both significant with the same direction and recovery fraction below 0.5, persistent-response; residual but not acute contrast significant, delayed-response; both significant with opposite directions, overshoot; both significant with recovery fraction at least 0.5, partial-recovering. “Fast-recovering” therefore denotes loss of FDR support at Time60 and does not prove complete normalization. Let a and r denote the Time0–Pre and Time60–Pre coefficients. Acute perturbation was |a|, residual baseline deviation (RBD) was |r|, and recovery fraction was 1 − |r|/|a|, with undefined ratios treated as missing. The scoring workflow bounded this fraction to [−1,1]. Higher values indicate a greater reduction in the initial displacement; negative values indicate a larger residual than acute displacement. Recovery-status categories and scoring cutoffs are specified in S1 Text.

### External intensity support in ST004303

ST004303 provided external exercise-related support under MIE and SIE conditions [32]. In both MIE and SIE, 1P denotes sampling before exercise, 2P immediately after exercise completion, and 4P 3 h after exercise completion. Within each treatment, models included time and participant fixed effects (~0 + time + subject); contrasts were 2P–1P, 4P–1P, and 4P–2P. Benjamini–Hochberg adjustment was applied separately to each contrast across all 266 retained features before restricting results to matched features. Direction support was defined permissively: the sign of any matched external contrast could agree with the discovery acute or residual contrast. This criterion was not restricted to temporally corresponding comparisons. Nominal support was p < 0.05 and FDR support was adjusted p < 0.05 in at least one matched record. EISI summarized differences in effect magnitudes and support counts between the two exercise conditions; it was not an interaction test. Differences in protocol and recovery duration preclude treating these results as direct replication of the 60-min endpoint.

### Staged candidate prioritization

A study-specific rule-based procedure prioritized candidates. CERS 2.0 combined discovery, kinetic, residual-deviation, external-support, and intensity-support components into a score from 0 to 18. Tiers 1–4 were defined by scores ≥12, 9–11, 6–8, and <6. ERMPS then awarded one point for each of five criteria: CERS Tier 1 or 2; large RBD or low bounded recovery fraction; external statistical support; an interpretable EISI support class; and biochemical-module interpretability. Scores of 5 and 4 defined high and moderate-to-high priority, respectively. EISI summarized relative MIE/SIE support, and IRCM combined EISI with ERMPS classes. These related scores reuse evidence and do not constitute independent validation. Exact component definitions, aggregation rules, and cutoffs are provided in S1 Text.

### Sensitivity analyses and software

Sensitivity analyses used nested relaxed, intermediate, and strict eligibility schemes. Their recovery criteria were RBD at or above the 50th, 75th, or 90th percentile, respectively, OR bounded recovery fraction at or below the 50th, 25th, or 10th percentile. All schemes required biochemical interpretability; successive schemes restricted CERS tier and external support as detailed in S1 Text. For the relaxed scheme, nominal external support could satisfy the evidence requirement even when neither recovery criterion was met. The recovery-fraction direction was corrected during revision to remain consistent with the primary ERMPS definition, in which lower values indicate poorer return toward baseline. Retention in all three schemes defined the threshold-stable subset. These analyses did not alter the primary 54-candidate set and did not assess sampling uncertainty. The original analyses were performed in R 4.5.1. The corrected threshold-sensitivity analysis and IRCM candidate classification were rerun in R. R scripts for the analysis workflow and figure generation are provided in S1 File.

### Ethics statement

This study involved secondary analysis of publicly available, de-identified human plasma metabolomics data. The ST001789 and ST004303 datasets were accessed for research purposes on 26 May 2026 and 30 May 2026, respectively. The authors did not recruit participants, conduct interventions, collect biospecimens, or have access to identifiable private information. Because the present study involved only secondary analysis of publicly available, de-identified data, the authors did not obtain additional ethics approval or participant consent for this analysis. Ethics approval and informed-consent procedures for the original data collection were handled by the original study investigators.

## Results

### ST001789 identifies baseline-referenced dynamic exercise-recovery responses

After preprocessing, ST001789 retained 455 metabolite features across 57 plasma samples collected before exercise, immediately after exercise, and after 60 min of recovery. Paired baseline-referenced analysis identified 198 metabolites that differed from the pre-exercise baseline immediately after exercise and 140 metabolites that remained different from baseline after 60 min of recovery. The Time60 versus Time0 contrast identified 116 metabolites with FDR-supported changes, indicating that metabolite levels continued to shift during the first 60 min of recovery. Together, these three contrasts captured the immediate exercise response, residual baseline deviation, and short-term recovery shift in ST001789. The results are summarized in Fig 2, with full paired-contrast outputs provided in S2 Table.

**Fig 2:**
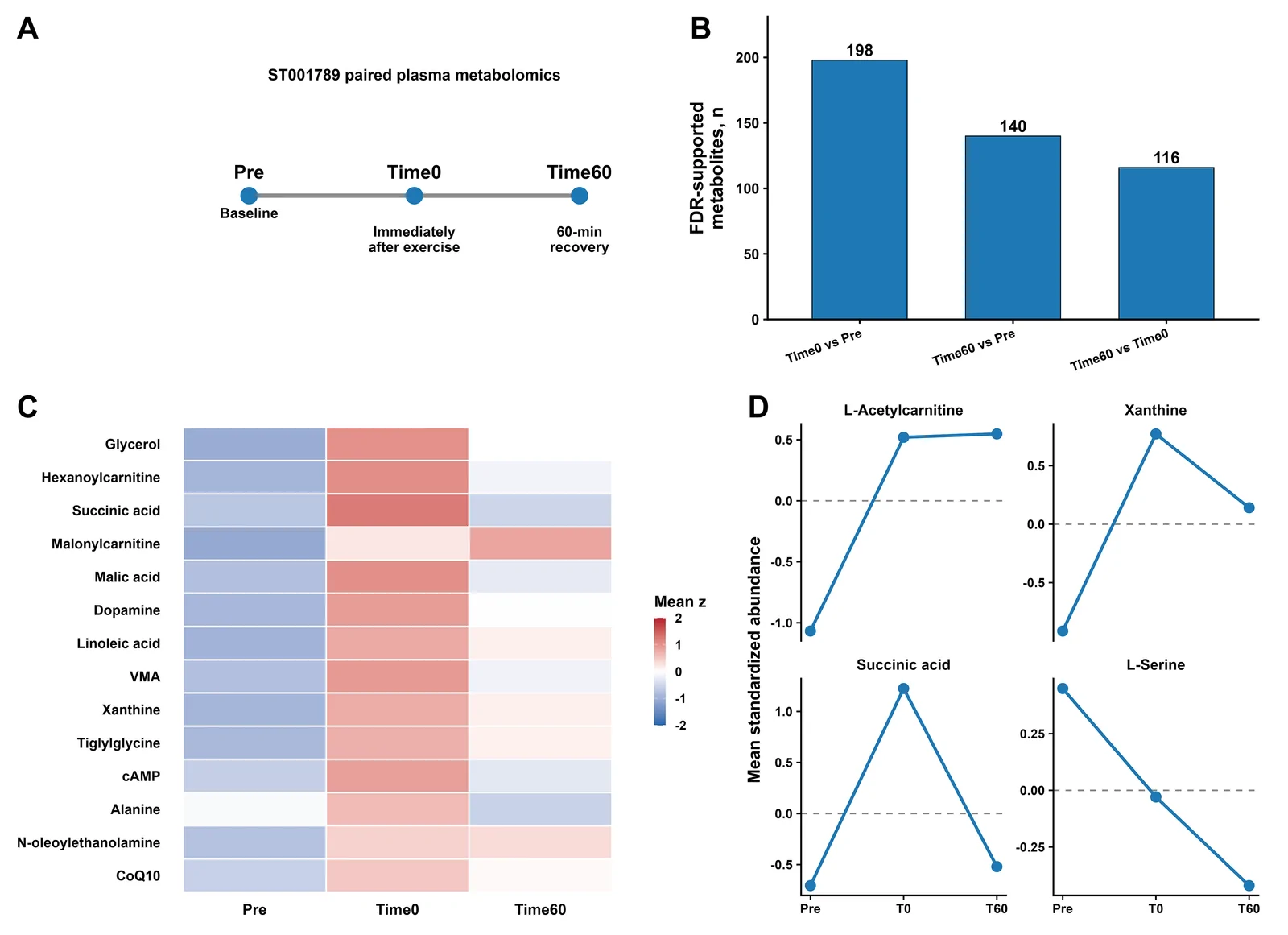
Baseline-referenced dynamic metabolite responses in ST001789. (A) Paired plasma metabolomics design with samples collected before exercise (Pre), immediately after exercise (Time0), and 60 min into recovery (Time60). (B) Numbers of FDR-supported metabolites in the Time0 versus Pre, Time60 versus Pre, and Time60 versus Time0 contrasts. (C) Heatmap of representative metabolites showing mean standardized abundance across the three time points. (D) Representative metabolite trajectories showing different early recovery behaviors, including persistent displacement, partial normalization, and continued deviation from baseline. FDR-supported metabolites were defined as adjusted p < 0.05. Abbreviations: FDR, false discovery rate; Pre, pre-exercise baseline; Time0, immediately after exercise; Time60, 60 min after exercise.

### Recovery-pattern classification reveals heterogeneous early recovery

Recovery-pattern classification separated the 455 metabolites according to their baseline-referenced time course. Non-responsive features were the largest group (n = 222), while 233 metabolites showed a detectable exercise-recovery pattern. Among the responsive metabolites, 93 were classified as fast-recovering, with an FDR-supported immediate post-exercise difference from baseline but no FDR-supported difference at Time60. Another 85 metabolites showed a persistent-response pattern, with deviation from baseline at both post-exercise time points. Delayed-response (n = 35), partial-recovering (n = 16), and overshoot features (n = 4) were less frequent, but they showed that some metabolites changed later, recovered incompletely, or moved beyond the initial post-exercise displacement. These results show that early metabolic recovery was not uniform, with fast-recovering, persistent-response, delayed-response, partial-recovering, and overshoot patterns coexisting within the 60-min recovery window.

### Residual baseline deviation and recovery efficiency highlight incomplete early normalization

Across the 455 features, mean absolute immediate perturbation was 0.497 and mean RBD at Time60 was 0.362, both in feature-specific z-score units. Recovery-status classification identified 59 high-recovery, 164 partial-recovery, 52 low-recovery, and 180 overshoot or delayed-divergence features. The acylcarnitine/fatty-acid oxidation module had a mean RBD of 0.889 across 34 features, indicating appreciable early residual deviation. The largest module mean, 1.074, belonged to lipid/fatty-acid signaling and represented only one feature, N-oleoylethanolamine. Module averages with very few members should not be taken as strong pathway-level evidence. Fig 3B–3D and S3 Table summarize these descriptive measures.

**Fig 3:**
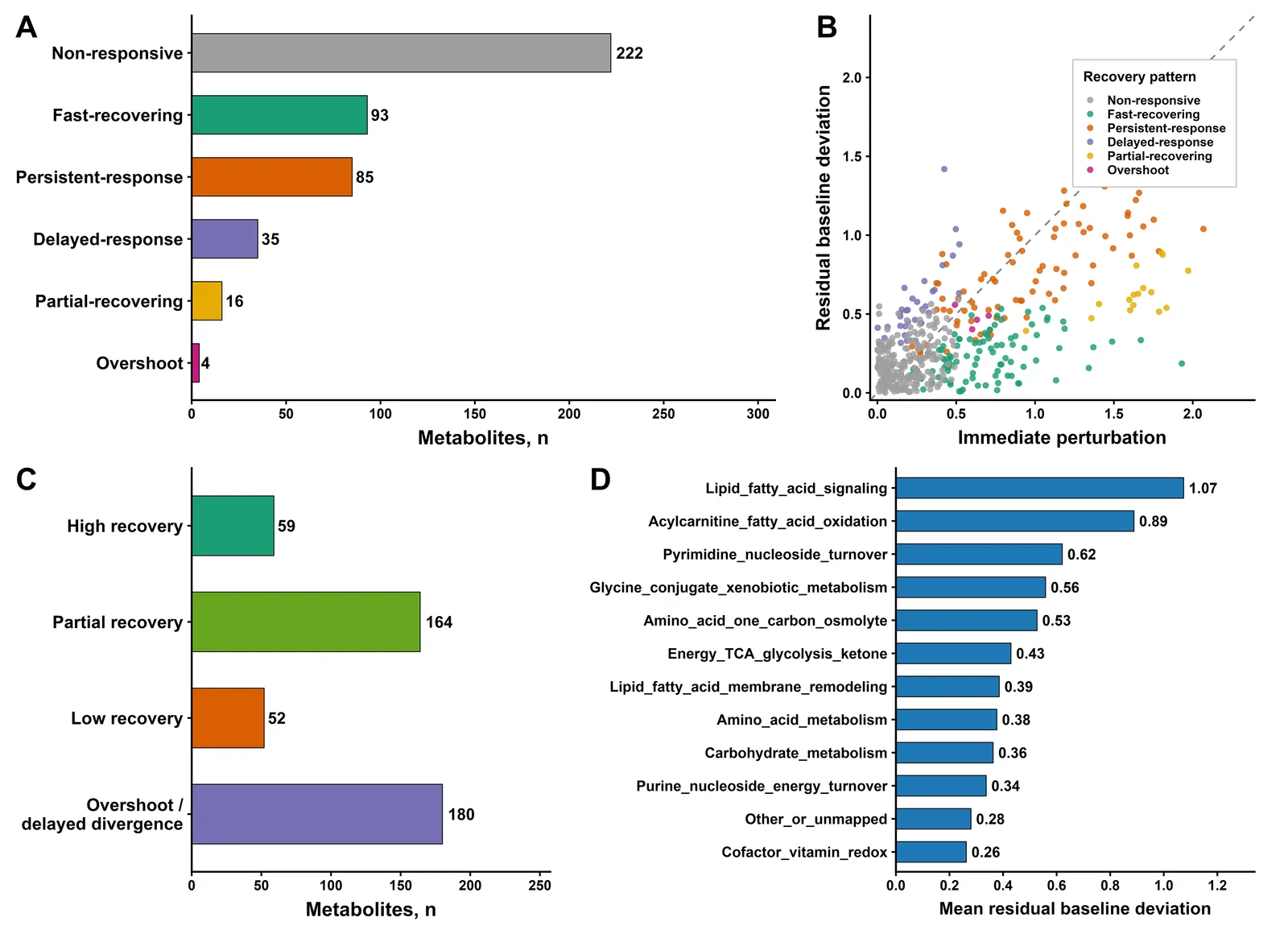
Recovery-pattern classification and residual baseline deviation in ST001789. (A) Distribution of recovery-pattern classes among the 455 retained metabolites. Metabolites were classified according to their paired changes across Pre, Time0, and Time60. (B) Scatter plot of immediate post-exercise perturbation and residual baseline deviation at 60 min, with points colored by recovery-pattern class. (C) Recovery-status categories based on the extent of normalization from Time0 to Time60. (D) Mean residual baseline deviation summarized by biochemical module. Residual baseline deviation indicates the remaining displacement at 60 min relative to the pre-exercise baseline. Abbreviations: Pre, pre-exercise baseline; Time0, immediately after exercise; Time60, 60 min after exercise; RBD, residual baseline deviation. Acute perturbation and RBD are expressed in z-score units.

### ST004303 provides external support for ST001789-derived metabolites

ST004303 was used to evaluate whether metabolites identified from ST001789 showed external support in an independent exercise dataset with moderate-intensity and sprint-interval conditions. Of the 455 discovery features from ST001789, 77 were matched to one or more external annotations. Among these matched metabolites, 74 had direction agreement in at least one external comparison with either discovery baseline contrast, and 66 had at least one FDR-supported external change. These counts do not require agreement at matching recovery times. These externally supported metabolites covered fatty-acid transport and oxidation, amino acid metabolism, purine/nucleoside turnover, lipid-related metabolism, redox/vitamin-related metabolism, and incompletely mapped features. The MIE and SIE conditions allowed the matched metabolites to be compared across exercise-intensity contexts, while ST001789 remained the primary discovery dataset. These external support results are summarized in Fig 4, with complete matched-metabolite results provided in S4 Table.

**Fig 4:**
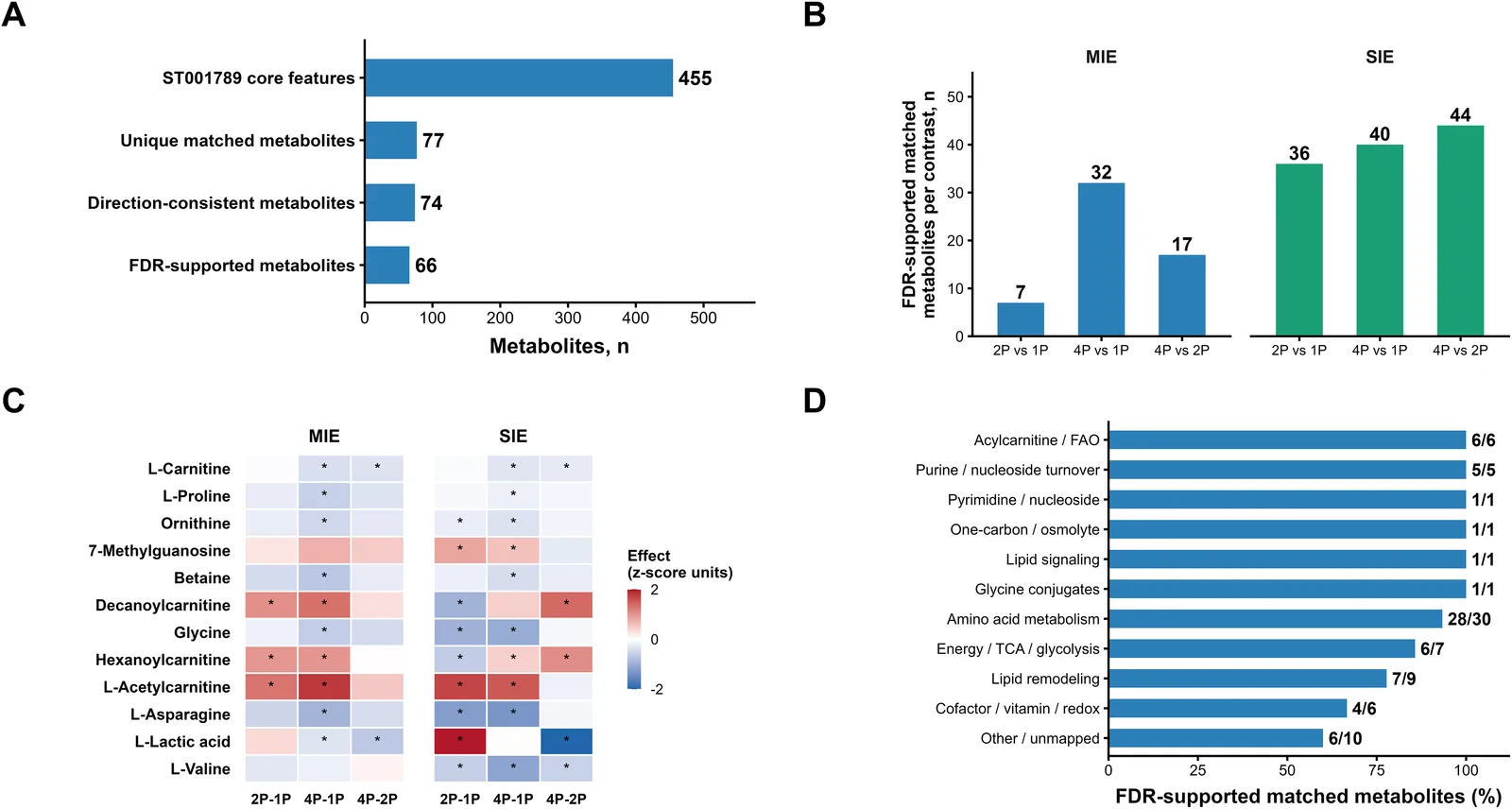
External support for ST001789 features in ST004303. (A) Counts of discovery features, matched features, and features with direction or FDR support in at least one external comparison. (B) FDR-supported matched discovery features, counted once per treatment/contrast. (C) Standardized mean differences for representative features, averaged across multiple mapped external features within each treatment/contrast. Asterisks indicate FDR < 0.05 for at least one mapped external feature; colors are capped at ±2 z-score units. (D) Percentage of matched discovery features with FDR support in each biochemical module; labels show supported/total counts. In both MIE and SIE, 1P denotes pre-exercise sampling, 2P immediately after exercise completion, and 4P 3 h after completion. Direction support in any external contrast does not establish replication at matched recovery times. MIE, moderate-intensity exercise; SIE, sprint-interval exercise; FDR, false discovery rate; FAO, fatty acid oxidation; TCA, tricarboxylic acid cycle.

### Staged consensus scoring ranks exercise-recovery metabolites

The staged consensus score integrated ST001789 discovery evidence, recovery-pattern class, residual baseline deviation, ST004303 external support, and intensity-related behavior to rank exercise-recovery metabolites. Across the 455 discovery features, 31 metabolites were assigned to Tier 1, 58 to Tier 2, 128 to Tier 3, and 238 to Tier 4. Tier 1 and Tier 2 metabolites were treated as higher-priority candidates because their scores incorporated recovery-pattern relevance, residual baseline deviation, external support, and interpretable exercise-intensity behavior. These higher-priority candidates covered fatty-acid transport and oxidation, amino acid metabolism, purine/nucleoside turnover, lipid and fatty-acid remodeling, and energy-related metabolism. The tier distribution is shown in Fig 5A, and complete scoring outputs are provided in S5 Table.

**Fig 5:**
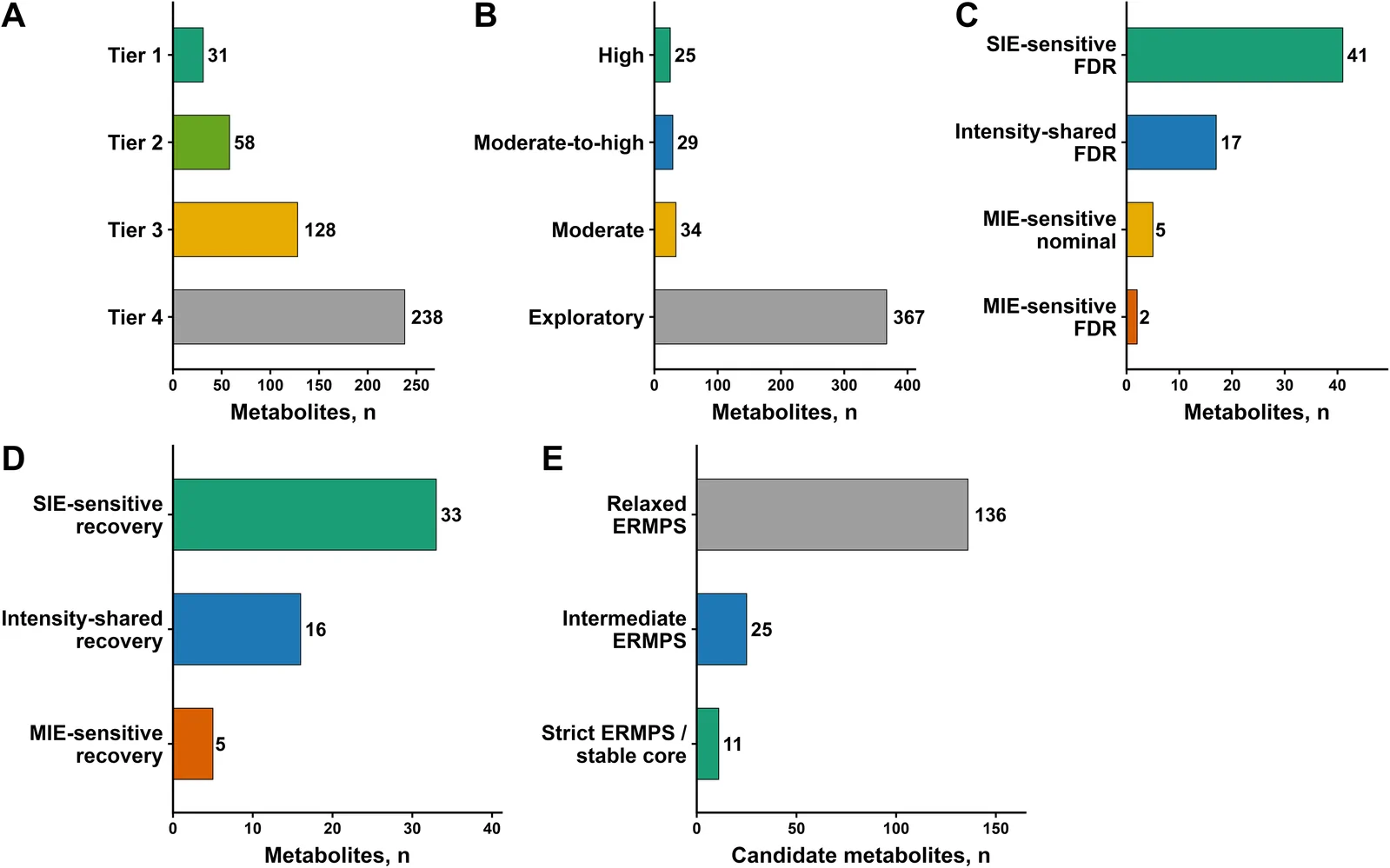
Candidate prioritization and threshold-stable metabolites. (A) Distribution of CERS 2.0 tiers among the 455 retained ST001789 metabolites. (B) Candidate-priority classes under the primary rule set. (C) Selected EISI categories among matched features; weak or non-prioritization categories are not shown. (D) IRCM groups combining candidate priority with exercise-intensity sensitivity among the 54 primary candidates. (E) Candidate counts under the corrected relaxed, intermediate, and strict sensitivity schemes (136, 25, and 11, respectively), with 11 features retained across all three schemes. FDR support indicates adjusted p < 0.05. Abbreviations: CERS 2.0, study-specific staged consensus exercise-recovery score; ERMPS, exercise-recovery metabolite prioritization score; EISI, exercise-intensity sensitivity index; IRCM, intensity–recovery coupling map; MIE, moderate-intensity exercise; SIE, sprint-interval exercise; FDR, false discovery rate.

### Staged prioritization identifies a 54-metabolite candidate set

The candidate-prioritization score was used to refine metabolites within the staged consensus tiers. Under the primary rule set, 25 metabolites were classified as high priority and 29 as moderate-to-high priority, yielding a 54-metabolite candidate set. For metabolites matched in ST004303, intensity-related support further grouped candidates into sprint-interval-sensitive, intensity-shared, and moderate-intensity-supported patterns. The combined prioritization map grouped the 54 candidates into 33 sprint-interval-sensitive, 16 intensity-shared, and 5 moderate-intensity-supported candidates. This step retained metabolites with both baseline-referenced recovery relevance and interpretable intensity-related behavior, producing a focused candidate set for future validation. Detailed ERMPS, EISI, and IRCM outputs are provided in S6 Table.

### Sensitivity analysis identifies candidates retained across nested schemes

After correcting the recovery-fraction direction, the relaxed, intermediate, and strict schemes retained 136, 25, and 11 metabolites, respectively. Eleven were retained across all three schemes, and 25 in at least two. These sets did not replace the primary 54-metabolite candidate set. Relative to the submitted sensitivity result, L-Methionine and Succinic acid left the strict subset, whereas L-isoleucine entered it. The revised core comprised fatty-acid transport and oxidation, amino acid metabolism, lipid signaling or remodeling, and purine/nucleoside turnover features. Retention reflects these nested rule definitions and should not be interpreted as independent validation. Fig 5E, Table 2, and S7 Table report the corrected analysis.

**Table 2:** Threshold-stable candidate metabolites for future validation.

Metabolite	Module	RBD	Recovery pattern / status	ERMPS class	EISI / IRCM class
L-Acetylcarnitine	Acylcarnitine / FAO	1.615	Persistent-response / Overshoot or delayed divergence	High priority	Intensity-shared (FDR); high-priority intensity-shared
L-Carnitine	Acylcarnitine / FAO	1.282	Persistent-response / Overshoot or delayed divergence	High priority	Intensity-shared (FDR); high-priority intensity-shared
Oleic acid	Lipid / fatty-acid membrane remodeling	1.269	Persistent-response / Low recovery	High priority	MIE-sensitive (FDR); high-priority MIE-sensitive
L-Asparagine	Amino-acid metabolism	0.815	Persistent-response / Overshoot or delayed divergence	High priority	SIE-sensitive (FDR); high-priority SIE-sensitive
L-Serine	Amino-acid metabolism	0.870	Delayed-response / Overshoot or delayed divergence	High priority	SIE-sensitive (FDR); high-priority SIE-sensitive
L-Valine	Amino-acid metabolism	0.828	Persistent-response / Low recovery	High priority	SIE-sensitive (FDR); high-priority SIE-sensitive
N-oleoylethanolamine	Lipid / fatty-acid signaling	1.074	Persistent-response / Low recovery	High priority	SIE-sensitive (FDR); high-priority SIE-sensitive
Xanthine	Purine / nucleoside energy turnover	1.055	Persistent-response / Partial recovery	High priority	SIE-sensitive (FDR); high-priority SIE-sensitive
Choline	Lipid / fatty-acid membrane remodeling	1.038	Delayed-response / Overshoot or delayed divergence	High priority	MIE-sensitive (FDR); high-priority MIE-sensitive
7-Methylguanosine	Purine / nucleoside energy turnover	0.871	Persistent-response / Partial recovery	High priority	SIE-sensitive (FDR); high-priority SIE-sensitive
L-isoleucine	Amino-acid metabolism	0.528	Delayed-response / Overshoot or delayed divergence	High priority	SIE-sensitive (FDR); high-priority SIE-sensitive

Note: The 11 metabolites are threshold-stable candidates retained across relaxed, intermediate, and strict ERMPS schemes. RBD is the absolute Time60–Pre coefficient in z-score units. Strict retention requires RBD ≥ its 90th percentile or bounded recovery fraction ≤ its 10th percentile, alongside Tier 1, biological interpretability, external FDR support, and an EISI class without weak support. ERMPS priority class summarizes candidate-prioritization status; High and Moderate-to-high indicate the corresponding ERMPS candidate classes. EISI / IRCM class summarizes intensity-related support and intensity–recovery coupling priority. FAO, fatty-acid oxidation; CERS 2.0, study-specific staged consensus exercise-recovery score; ERMPS, exercise-recovery metabolite prioritization score; EISI, exercise-intensity sensitivity index; IRCM, intensity–recovery coupling map; MIE, moderate-intensity exercise; SIE, sprint-interval exercise; FDR, false discovery rate.

## Discussion

This study performed a secondary analysis of public human plasma metabolomics data to characterize baseline-referenced metabolic responses during early post-exercise recovery. In ST001789, endurance exercise induced an immediate plasma metabolic response, with many metabolites still displaced from the pre-exercise baseline after 60 min of recovery. Recovery-pattern classification showed heterogeneous early recovery, including fast-recovering, persistent-response, delayed-response, partial-recovering, and overshoot features. In ST004303, matched metabolites showed exercise-related and intensity-related support under moderate-intensity and sprint-interval exercise conditions. The staged prioritization procedure organized the discovery set into a 54-metabolite candidate set and an 11-metabolite subset retained across the corrected sensitivity schemes. Together, these findings suggest that acute change, baseline return, residual deviation, cross-dataset support, and intensity-related behavior can be combined to organize early post-exercise metabolomic responses. The identified metabolites should be interpreted as hypothesis-generating candidates for future validation, not as validated recovery biomarkers.

### Baseline-referenced profiling separates acute response from residual deviation

In sport and exercise settings, recovery assessment is relevant to subsequent exercise loading because it places biological responses within a practical recovery context [34,35]. Because recovery is difficult to capture with a single measure, current approaches often combine training-load data, perceived recovery, wellness scores, neuromuscular tests, heart-rate-derived variables, and selected blood markers [36–39]. By using the pre-exercise baseline as a reference, this analysis separated metabolites with immediate post-exercise changes from those that still showed residual deviation after 60 min of recovery. This distinction is relevant because an acute metabolic change may reflect the immediate response to exercise, while residual baseline deviation points to incomplete early normalization. The identified metabolites should therefore be viewed as candidates that may complement existing recovery-assessment measures, not as stand-alone indicators of recovery status.

### Residual baseline deviation indicates incomplete early normalization

Residual baseline deviation at 60 min should be interpreted as indicating incomplete early normalization, not as a measure of overall recovery. Exercise recovery can extend over hours or days, and neuromuscular performance, biochemical markers, soreness, perceived fatigue, and sport-specific performance often recover at different rates [40,41]. These time courses also depend on exercise mode, load characteristics, recovery strategy, and the outcome being assessed [42,43]. Within this early window, the 60-min baseline-referenced contrast identified metabolites that still deviated from their pre-exercise levels. These metabolites may add information beyond the immediate post-exercise contrast because they reflect continued baseline deviation, not only the initial perturbation. Residual baseline deviation should therefore not be treated as a direct readiness marker; instead, it identifies candidates that require testing at later recovery time points and against functional recovery outcomes.

### Intensity-related patterns inform recovery-candidate interpretation

The intensity-related categories were used to interpret candidate metabolites in the context of exercise load, not to rank exercise intensities. High-intensity interval and repeated-sprint sessions provide strong training stimuli, but they can also impose substantial physiological, neuromuscular, perceptual, and performance demands [44,45]. Sprint-based exercise can acutely affect neuromuscular function, and HIIT programming requires a balance between training load, recovery, and adaptation [46,47]. Accordingly, sprint-interval-sensitive candidates may be relevant for future testing after sessions with high internal stress, while intensity-shared candidates may reflect recovery features that are less specific to one exercise format. Moderate-intensity-supported candidates also remain relevant for future testing because recovery demand depends not only on exercise intensity but also on exercise mode, accumulated load, repeated-effort requirements, and between-bout recovery time [48].

### Candidate metabolite sets should complement existing recovery-assessment measures

The metabolite set identified in this study should be viewed as a prioritization result, not as a ready-to-use monitoring test. In sport and exercise settings, biomarker interpretation is strengthened when markers are assessed repeatedly and alongside contextual information and complementary physiological domains [49,50]. Blood- and saliva-based recovery markers can also be influenced by sport discipline, sampling time, training status, and the biological matrix being assessed [51,52]. The 54-metabolite candidate set and the 11 candidates retained across all corrected sensitivity schemes therefore provide a focused starting point for validation and should be tested alongside established recovery-assessment measures. Their future value will depend on whether they add useful information to systems that already integrate load, wellness, performance, and health-related data [53].

### Threshold-stable candidates cover multiple recovery-relevant domains

The 11 candidates retained across the corrected sensitivity schemes form a compact group for further testing. L-acetylcarnitine and L-carnitine belonged to the fatty-acid transport and oxidation domain, which has been linked to post-exercise lipid handling and recovery-related metabolic variation [54,55]. The amino acid-related candidates, including L-asparagine, L-serine, L-valine, and L-isoleucine, are consistent with evidence that amino acid profiles vary with training phase, substrate redistribution, and recovery-related adaptation in endurance athletes [56,57]. Xanthine and 7-methylguanosine mapped to purine/nucleoside turnover, whereas choline, oleic acid, and N-oleoylethanolamine linked the set with lipid-related remodeling or signaling. Exercise studies have also reported changes in amino acids, acylcarnitines, and lipid-related metabolites after training or exercise-based interventions [58]. These broad biological associations motivate joint testing but do not establish that the candidates measure functional recovery. Their retention also depends on the selected scoring rules and the biochemical-interpretability criterion.

### Candidate metabolites require validation against practical recovery outcomes

Validation should establish both measurement reliability and relevance to functional recovery. Candidate identities should first be confirmed using authentic standards, chromatographic retention, and MS/MS evidence, with attention to unresolved isomers. Targeted assays should assess calibration, precision, matrix effects, sample stability, and quantification limits before longitudinal testing. Larger independent cohorts should then use repeated baseline and post-exercise sampling under standardized dietary, fasting, hydration, and collection-time conditions. Sampling should extend beyond the early recovery window and accompany perceived recovery, autonomic measures, neuromuscular performance, and repeated-bout exercise outcomes [59–63]. Prespecified analyses should test whether individual metabolites or a reduced candidate panel explain these outcomes beyond exercise load and established monitoring measures. This would distinguish reproducible exercise responses from measurements that add useful information about recovery.

### Sampling timing and individual baselines are required for interpretation

Individual baselines help interpret metabolite changes because starting levels and exercise responses vary with age, sex, fitness, and training history [64,65]. Within-participant comparisons reduce the influence of stable between-person differences, but do not remove changes in nutritional exposure, hydration, or plasma volume during an exercise session. Fasting status and clock time also affect comparisons with baseline. These factors can contribute to apparent persistence or normalization and may differ between the two source studies. Future validation should therefore combine repeated individual reference measurements [66] with standardized sampling, processing, and quality control [67]. External support in the present analysis should be interpreted within each study protocol, without attributing all between-protocol differences to exercise intensity.

### Interpretation boundaries and validation requirements

Both datasets were small: ST001789 included 19 participants, and ST004303 included 19 participants divided between MIE (n = 9) and SIE (n = 10). The 57 repeated samples in each dataset do not constitute 57 independent participants. These sample sizes limit precision and the assessment of heterogeneity by age, sex, and fitness. Candidate selection may also be sensitive to individual participants. Retention across nested scoring schemes describes dependence on those rules; it is not independent replication, resampling-based stability, or evidence of predictive accuracy. The candidate set therefore remains hypothesis-generating.

The studies also differed in sex composition, exercise protocol, recovery duration, analytical platform, and metabolite coverage. Only 77 of the 455 discovery features were matched, so external support pertains to this overlapping subset. Both studies analyzed plasma, but using the same sample matrix does not eliminate differences in collection conditions or annotation confidence. Library-based annotations may retain structural ambiguity, particularly for isomers and lipid species. The secondary analysis does not independently establish the molecular identity of every matched feature. Removing stereochemical prefixes and retaining one-to-many matches can overstate feature-level agreement and disproportionately weight multiply matched annotations. Module-based eligibility can favor already interpretable metabolites. No formal treatment-by-time test established that response differences were attributable to intensity.

Residual baseline deviation describes a measured biochemical state; it does not establish impaired performance or readiness for another exercise bout. The present analysis did not validate the candidate set against functional recovery outcomes. Standardized assays and larger prospective cohorts are needed to assess analytical reliability, reproducibility, and added value in the intended recovery-monitoring setting [68–71].

### Toward multimodal recovery assessment

The candidate metabolites should be viewed as one possible biochemical component of multimodal recovery assessment, not as stand-alone laboratory results. Repeated assessment data are most useful when interpreted in the practical context in which coaches, practitioners, and athletes manage day-to-day training [72]. Wearable sensors and mobile heart-rate-variability tools can provide frequent, low-burden information on autonomic status, workload, and movement-related responses, whereas metabolite measurements may provide periodic biochemical information when blood sampling is feasible [73,74]. This broader context is important because recovery assessment is shaped not only by physiology but also by athlete preference, coaching context, competition schedule, and feasibility [75]. The candidate set identified here should therefore be evaluated for added value alongside field-based measures and athlete-reported indicators before any decision-support role is considered.

## Conclusions

This study used public human plasma metabolomics data to characterize baseline-referenced metabolic responses during early post-exercise recovery. In ST001789, many metabolites had not returned to the pre-exercise baseline after 60 min of recovery, indicating that early metabolic normalization was incomplete. In ST004303, matched metabolites showed exercise-related and intensity-related support under moderate-intensity and sprint-interval exercise conditions. By combining recovery pattern, residual baseline deviation, external support, intensity-related behavior, and threshold stability, the analysis prioritized a 54-metabolite candidate set and an 11-metabolite threshold-stable core. These metabolites should be interpreted as hypothesis-generating candidates. Their value should be tested in longitudinal studies that include standardized sampling, individual baselines, repeated measures, and practical recovery outcomes.

## Supporting information

S1 Text Scoring and candidate-prioritization rules. (DOCX)

S1 Table Dataset preprocessing and quality-control summary. (XLSX)

S2 Table Paired baseline-referenced metabolite responses in ST001789. (XLSX)

S3 Table Recovery-pattern classification, residual baseline deviation, and recovery efficiency in ST001789. (XLSX)

S4 Table External support analysis of ST001789-derived metabolites in ST004303. (XLSX)

S5 Table Full CERS 2.0 scoring results. (XLSX)

S6 Table ERMPS, EISI, and IRCM candidate-prioritization results. (XLSX)

S7 Table ERMPS threshold sensitivity and stability analysis. (XLSX)

S1 Fig Volcano plots of paired baseline-referenced metabolite responses in ST001789. (A) Time0 versus Pre, (B) Time60 versus Pre, and (C) Time60 versus Time0 contrasts. Each point represents one retained metabolite. The x axis is in feature-specific z-score units. Colored points indicate FDR-supported metabolites, and grey points indicate non-FDR-supported metabolites. The horizontal dashed line indicates FDR = 0.05, and the vertical dotted line indicates standardized mean difference = 0. FDR, false discovery rate; Pre, pre-exercise baseline; Time0, immediately after exercise; Time60, 60 min into recovery.
(TIF)

S2 Fig Recovery fraction, residual baseline deviation, and recovery-status composition by recovery-pattern class. (A) Recovery fraction and (B) residual baseline deviation across recovery-pattern classes. (C) Recovery-status composition within each recovery-pattern class. Points represent individual metabolites, and boxplots summarize the median and interquartile range. Recovery fraction indicates the proportion of the immediate perturbation that returned toward baseline by Time60. Residual baseline deviation indicates the remaining absolute displacement from baseline at Time60.
(TIFF)

S3 Fig External intensity-support analysis of ST001789-derived metabolites in ST004303. (A) Counts of matched discovery features with nominal (p < 0.05) or FDR support (adjusted p < 0.05), counted once per treatment/contrast. (B) Percentages of the 77 matched discovery features showing direction agreement with the ST001789 acute (Time0–Pre) or residual (Time60–Pre) contrast. (C) Maximum acute and residual support scores across matched external records; colors indicate combinations of FDR and direction support. (D) Percentage of matched discovery features with FDR support in each biochemical module; labels show supported/total counts. In both MIE and SIE, 1P denotes pre-exercise sampling, 2P immediately after exercise completion, and 4P 3 h after completion. Direction agreement does not imply replication at the same recovery time point. MIE, moderate-intensity exercise; SIE, sprint-interval exercise; FDR, false discovery rate; FAO, fatty acid oxidation; TCA, tricarboxylic acid cycle.
(TIF)

S4 Fig ERMPS threshold-sensitivity and stability analysis under relaxed, intermediate, and strict ERMPS schemes. (A) Candidate counts under the corrected relaxed, intermediate, and strict schemes (136, 25, and 11, respectively). (B) Numbers of features retained in all three, exactly two, exactly one, or no schemes. (C) Retention rates under each scheme among the 12 and 20 highest CERS-ranked features, with ties ordered as in S5 Table. These ranking subsets are distinct from the 11-feature stable core. (D) Residual baseline deviation in z-score units for the 11 features retained across all three schemes. Stability across these nested selection rules does not constitute independent validation. ERMPS, exercise-recovery metabolite prioritization score; CERS, study-specific staged consensus exercise-recovery score.
(TIF)

S1 File R scripts for the analysis workflow, corrected threshold-sensitivity analysis, IRCM candidate classification, and figure generation. (ZIP)
