# Supplementary material for: Baseline-referenced plasma metabolomic trajectories during early post-exercise recovery: A secondary analysis of public human exercise metabolomics data
Source: PLoS One. 2026 Oct 30;21(10):e0361757. doi: 10.1371/journal.pone.0361757 (PMC13722211; doi:10.1371/journal.pone.0361757)
Supplement: Supplementary file 1 [file pone.0361757.s001.docx]

**S1 Text. Scoring and candidate-prioritization rules.**

Candidate prioritization used a prespecified rule-based framework applied uniformly to all retained metabolites. CERS 2.0 combined five evidence domains: discovery evidence in ST001789 (0–3), recovery-pattern/kinetic behavior (0–4), residual baseline deviation (RBD; 0–3), external support in ST004303 (0–4), and intensity-related behavior (0–4). The total CERS 2.0 score was calculated as the sum of these domains and classified as Tier 1 (≥12), Tier 2 (9–11), Tier 3 (6–8), or Tier 4 (0–5).

**Effect units and recovery measures**

Both datasets were modeled after log2 transformation and feature-wise z standardization. The limma output field named logFC therefore contains standardized mean differences, not log2 fold changes. Let a = Time0–Pre and r = Time60–Pre. Acute perturbation A = |a|; RBD = |r|; the signed recovery delta is Time60–Time0. The recovery fraction F = 1 − RBD/A is missing when the ratio is undefined. CERS and ERMPS use F bounded to [−1, 1], denoted Fc. Larger values indicate more baseline return. A negative value means RBD exceeds A; it does not on its own show that the trajectory crossed baseline. Normalized RBD is RBD/(A + 10⁻⁶).

Recovery-status labels use unbounded F: high recovery, F ≥ 0.75; partial recovery, 0.25 ≤ F < 0.75; low recovery, 0 ≤ F < 0.25; overshoot or delayed divergence, F < 0. The low-acute-perturbation label takes precedence when A < 10⁻⁶ or A is missing. Trajectory classes instead use significance and sign rules specified in Methods. In particular, the trajectory label overshoot and the broader negative-F status are not interchangeable.

**Cross-dataset normalization and aggregation**

Matching used exact agreement between normalized feature-name or RefMet-name keys. The implementation converted names to lower case, removed “(low)” and “(high)”, replaced underscores with spaces, collapsed whitespace, and removed characters outside its specified alphanumeric and punctuation set. It also removed leading L-, D-, DL-, and optical-rotation prefixes and applied an explicit synonym dictionary covering selected organic acids and carnitines. Duplicate feature–key pairs were removed before joining. Each discovery-feature/external-feature/treatment/contrast combination was counted once, but one-to-many feature mappings were retained and aggregated. S4 Table lists the reported matches and annotation flags. This permissive procedure matches reported annotations and does not confirm equivalent stereochemistry or resolve lipid isomers. For example, L-Carnitine matched both D-Carnitine and L-Carnitine, and L-Tryptophan matched D-(+)-Tryptophan.

The allowed key characters are lower-case letters, digits, colon, semicolon, comma, plus, hyphen, whitespace, parentheses, and square brackets. The implementation removes leading L-, D-, DL-, L or D followed by a space, and optical-rotation markers (+) and (−). The explicit synonym dictionary is applied after this normalization: pyruvic acid → pyruvate; lactic acid → lactate; malic acid → malate; succinic acid → succinate; fumaric acid → fumarate; citric acid → citrate. Oxoglutaric acid, 2 oxoglutaric acid, 2-oxoglutaric acid, alpha ketoglutaric acid, alpha-ketoglutaric acid, 2 oxoglutarate, 2-oxoglutarate, alpha ketoglutarate, and alpha-ketoglutarate map to oxoglutarate. 3 hydroxybutyric acid, 3-hydroxybutyric acid, beta hydroxybutyrate, beta-hydroxybutyrate, and bohb map to 3-hydroxybutyrate. Acetyl carnitine, acetylcarnitine, and acetyl-carnitine map to acetylcarnitine; palmitoyl carnitine and oleoyl carnitine map to palmitoylcarnitine and oleoylcarnitine. Other names are unchanged after normalization. No fuzzy matching or new spectral verification was performed. Lipid matches follow these same name/RefMet rules; acyl-chain positions and unresolved isomers are not inferred.

Empty/NA keys are discarded. Names and RefMet fields provide alternative keys for each feature. Duplicate feature–key pairs are removed before joining, and duplicate discovery-feature/external-feature/treatment/contrast records are removed afterward. Distinct external feature matches are retained. Means, minima, support counts, and maxima below use these retained records, so one-to-many matches can contribute more than one record per contrast. The 77 matched units are discovery features, not 77 structurally confirmed molecular correspondences. Feature-count summaries in Fig 4 and S3 Fig count each discovery feature once per treatment/contrast, using any qualifying matched record; the denominator for each direction-consistency proportion is 77.

**CERS 2.0 components**

Each criterion below contributes one point. Discovery score (0–3): a trajectory other than non-responsive; acute FDR < 0.05; residual FDR < 0.05. Kinetic score (0–4): recovery-shift FDR < 0.05; partial, low, or overshoot/delayed-divergence recovery status; A at least its median; and RBD at least its median. RBD score (0–3): both A and RBD at least their medians; normalized RBD at least its median; and Fc < 0.25.

For each matched external record, auxiliary acute and residual support scores (each 0–6) award points for a responsive discovery trajectory; significance of the corresponding discovery baseline contrast; agreement between its sign and the external coefficient; external nominal p < 0.05; external FDR < 0.05; and external absolute standardized coefficient ≥0.5. Every external contrast is compared with both discovery baseline contrasts. Maximum support scores refer to the maximum across all matched external records.

External-support score (0–4): at least one match; any external FDR < 0.05; any acute or residual sign agreement; and a maximum auxiliary acute or residual support score ≥5. Within each treatment–contrast group, intensity support requires any FDR-supported record and any acute or residual sign agreement; these can arise from different matched records. Intensity-support score (0–4) awards one point each for any supported SIE group, any supported MIE group, support in both treatments, and at least two supported treatment–contrast groups. CERS 2.0 is the sum (0–18). Tier 1 is ≥12, Tier 2 is 9–11, Tier 3 is 6–8, and Tier 4 is <6.

**EISI and primary ERMPS**

EISI summarized intensity-related support in ST004303. For each matched discovery feature and treatment, calculate the mean absolute standardized coefficient and the numbers of matched records with nominal p < 0.05 and FDR < 0.05. Let D be the SIE minus MIE mean absolute coefficient. EISI applies these ordered rules: (1) both treatments have FDR support and |D| < 0.10, intensity-shared FDR support; (2) more SIE FDR records and D > 0, SIE-sensitive FDR support; (3) more MIE FDR records and D < 0, MIE-sensitive FDR support; (4) SIE-only FDR support, SIE-supported; (5) MIE-only FDR support, MIE-supported; (6) more SIE nominal records and D > 0, SIE-sensitive nominal tendency; (7) more MIE nominal records and D < 0, MIE-sensitive nominal tendency; (8) both treatments have FDR support, intensity-shared FDR support; otherwise, weak or comparable support. The auxiliary intensity-strength ordering is |D| + 0.20 × |SIE FDR count − MIE FDR count|. These are descriptive rules, not formal tests of between-treatment differences.

ERMPS (0–5) awards one point for each of: Tier 1/2; RBD ≥Q75(RBD) OR Fc ≤Q25(Fc); any external FDR support OR at least two nominally supported external records; one of the seven named supported EISI classes above; and a nonmissing interpretable module. The module rule accepts a nonmissing module containing Acylcarnitine, fatty_acid, Energy, TCA, glycolysis, ketone, Amino_acid, one_carbon, osmolyte, Purine, nucleoside, nucleotide, Lipid, membrane, redox, Pyrimidine, or xenobiotic (case-insensitive), or any nonmissing module other than Other_or_unmapped. ERMPS translated CERS 2.0 evidence into candidate-prioritization classes: high priority, score 5; moderate-to-high priority, score 4; moderate priority, score 3; and exploratory priority, scores 0–2. The primary candidate set consists of scores ≥4 (54 features).

**Corrected sensitivity schemes**

Threshold sensitivity was assessed using relaxed, intermediate, and strict schemes. The relaxed scheme retained a broad candidate set, the intermediate scheme retained candidates with more consistent support, and the strict scheme retained the most conservative candidates. Metabolites retained across all three schemes were defined as the threshold-stable core; those retained in at least two schemes were considered partially stable candidates.

The relaxed scheme requires Tier 1–3 and biological interpretability, plus either any nominal external support or [RBD ≥Q50(RBD) OR Fc ≤Q50(Fc)]. The intermediate scheme requires Tier 1–2, biological interpretability, any external FDR support, and [RBD ≥Q75(RBD) OR Fc ≤Q25(Fc)]. The strict scheme requires Tier 1, biological interpretability, any external FDR support, a nonmissing EISI class without “weak”, and [RBD ≥Q90(RBD) OR Fc ≤Q10(Fc)]. These nested schemes yield 136, 25, and 11 features. Eleven occur in all three, 14 in exactly two, 111 in exactly one, and 319 in none. The “top 12” and “top 20” CERS ranking subsets shown in S4 Fig are separate from this 11-feature intersection.

Percentiles are calculated across all 455 retained discovery features using R’s default quantile definition (type 7), excluding missing values. Numerical cutoffs from full-precision data are Q50/Q75/Q90(RBD) = 0.2817620172/0.4964645828/0.8090065561, and Q50/Q25/Q10(Fc) = 0.2355920484/−0.5831436799/−1. The lower-tail cutoff reaches the −1 bound in the strict scheme; the analysis is therefore partly sensitive to this clamping choice. The submitted script had used Fc ≥Q50/Q75/Q90, producing 137/36/12. This was corrected because high Fc denotes better return, unlike high RBD. The revision does not alter the primary ERMPS scores or the 54-candidate set.

**IRCM and interpretation**

IRCM combined ERMPS class with EISI behavior to assign high-priority SIE-sensitive, MIE-sensitive, or intensity-shared candidate groups. IRCM groups ERMPS scores 4–5 by SIE-sensitive/supported, MIE-sensitive/supported, intensity-shared, or weak EISI support. The 54 primary candidates comprise 33 SIE-sensitive, 16 intensity-shared, and 5 MIE-sensitive/supported features. Other ERMPS priorities remain exploratory. CERS, ERMPS, EISI, and IRCM share inputs; their agreement must not be counted as independent evidence. Threshold retention does not establish molecular identity, functional recovery relevance, generalizability, or robustness to participant resampling.
